# Supplementary material for: WordCommentsAnalyzer: A windows software tool for qualitative research
Source: F1000Res. 2018 Sep 4;7:536. Originally published 2018 May 3. [Version 2] doi: 10.12688/f1000research.14819.2 (PMC6137411; doi:10.12688/f1000research.14819.2)
Supplement: Supplementary file 3 [file f1000research-7-17614-s0002.tgz › b2a79496-4ada-47c4-9682-a99ce867836d.zip!/Eight Iranian Health Journals/Journal of Research in Rehabilitation Sciences _9.docx]

© Journal of Research in Rehabilitation Sciences . This work is licensed under CC BY-NC 4.0 (https://creativecommons.org/licenses/by-nc/4.0/).

Volume & Issue: دوره 12، شماره 2: 1395

Title: بررسی رابطه قوس‌های کمری و پشتی با سابقه زمین خوردن‏، تعادل و ترس از زمین خوردن در سالمندان

http://jrrs.mui.ac.ir/index.php/jrrs/article/view/2576

مقدمه: یکی از مشکلات سالمندان زمین خوردن می‌باشد که عوارض مختلفی از جمله شکستگی لگن، ضربه به سر و حتی مرگ به دنبال دارد. تغییرات در قوس‌‌های کمری و پشتی نیز در میان سالمندان شایع است. مطالعات محدودی به بررسی رابطه بین میزان قوس کمری با تعادل پرداختند. برخی محققان افزایش کایفوز را عامل کاهش تعادل عنوان نمودند؛ در حالی که برخی دیگر، وجود رابطه بین این دو متغیر را تأیید نکردند. مطالعه حاضر به بررسی رابطه بین قوس‌‌های ستون فقرات کمری و پشتی با تعادل و ترس از زمین خوردن در سالمندان پرداخت. مواد و روش‌ها: این مطالعه بر روی 47 سالمند سالم زن و مرد صورت گرفت. در این پژوهش قوس‌های پشتی و کمری توسط خط‌کش منعطف اندازه‌گیری شد. سپس رابطه بین قوس‌های‌ کمری و پشتی با تعادل در سالمندان از طريق بررسي نتايج آزمون‌هاي Berg (Berg balance scale) و برخاستن و راه رفتن (Timed up and go یا TUG)، بررسی گردید. پرسش‌نامه FES-I (Fall efficacy scale-international) نیز برای بررسی ترس از زمین خوردن مورد استفاده قرار گرفت. یافته‌ها: در آزمون Pearson، ارتباط معنی‌دار بین نمرات آزمون‌های Berg و TUG و نیز نمره پرسش‌نامه FES-I با میزان قوس‌ ستون فقرات در ناحیه کمر و پشت مشاهده شد. نتیجه‎گیری: نتایج این مطالعه نشان داد که بین قوس‌های‌ ستون فقرات پشتی و کمری با تعادل و ترس از زمین خوردن در سالمندان ارتباط وجود دارد

Title: تغییرات پارامترهای فضایی- زمانی راه رفتن در سالمندان به دنبال تکالیف مختلف حافظه کاری

http://jrrs.mui.ac.ir/index.php/jrrs/article/view/2604

مقدمه: مطالعات پیشین نشان می‌دهد که الگوی راه رفتن سالمندان تحت تأثیر تکلیف شناختی همزمان قرار می‌گیرد. هرچند هنوز مشخص نیست که با فعال شدن زیرسیستم‌های مختلف حافظه کاری، الگوی راه رفتن چگونه تحت تأثیر قرار می‌گیرد. هدف از انجام پژوهش حاضر، تأثیر نوع تکلیف حافظه کاری بر پارامترهای فضایی- زمانی الگوی راه رفتن بود. مواد و روش‌ها: 12 سالمند (با میانگین سنی 19/2 ± 5/67) به صورت در دسترس انتخاب و تحت 3 شرایط آزمایشی راه رفتن به شکل مجرد، راه رفتن همزمان با فعال شدن لوح دیداری- فضایی حافظه کاری و راه رفتن همزمان با فعال شدن حلقه واج‌شناختی حافظه کاری مورد آزمون قرار گرفتند. شرکت کنندگان 12 کوشش راه رفتن را در هر شرایط اجرا کردند که از بین آن‌ها 6 کوشش انتخاب شد. ملاک انتخاب این 6 کوشش در شرایط تکلیف دوگانه، کوشش‌هایی بود که در آن شرکت کننده پاسخ صحیح به تکلیف حافظه کاری داده بود. در شرایط راه رفتن مجرد این 6 کوشش به صورت تصادفی انتخاب شد. کوشش‌های راه رفتن توسط دستگاه Motion analysis ثبت شد و داده‌های مرتبط با الگوی راه رفتن با استفاده از تحلیل واریانس با اندازه‌های تکراری تحلیل شد. یافته‌ها: بین 3 شرایط مختلف آزمایشی در سرعت راه رفتن (05/0 > P، 54/64 = (2،142)F)، طول گام (05/0 > P، 82/45 = (2،142)F) و عرض گام (05/0 > P، 36/16 = (2،142)F) تفاوت معنی‌داری وجود داشت. نتیجه‎گیری: تکلیف دیداری- فضایی در مقایسه با تکلیف واج‌شناختی منجر به طول گام کوتاه‌تر، عرض گام بیشتر و سرعت راه رفتن کمتر شد و تداخل بیشتری در راه رفتن ایجاد کرد. به نظر می‌رسد که برای کنترل راه رفتن و پردازش دیداری- فضایی، شبکه‌های عصبی مشترکی وجود دارد. از این‌رو، مداخلات تکلیف دوگانه دیداری- فضایی می‌تواند موجب بهبود راه رفتن سالمندان شود.

Title: درد، آستانه درد فشاری و ناتوانی افراد دارای نقاط ماشه‌ای فعال عضله تراپزیوس فوقانی پس از یک جلسه سوزن زدن خشك

http://jrrs.mui.ac.ir/index.php/jrrs/article/view/2605

مقدمه: سندرم درد مایوفاسیال (Myofascial pain syndrome یا MPS) یک مشکل شایع و یکی از اختلالات غیر مفصلی اسکلتی- عضلانی است که با درد موضعی و ارجاعی در اثر نقاط ماشه‌ای فعال در بافت عضله مشخص می‌شود. هدف از انجام مطالعه حاضر، بررسی اثرات یک جلسه سوزن زدن خشك بر افراد داراي نقاط ماشه‌اي فعال عضله تراپزیوس فوقاني بود. مواد و روش‌ها: 23 فرد مبتلا به نقطه ماشه‌اي فعال عضله تراپزیوس فوقاني وارد مطالعه شدند. داده‌هاي شدت درد بر اساس مقیاس خطی- دیداری (Visual Analogue Scale یا VAS) و شاخص ناتوانی فرد بر اساس امتیاز نسخه فارسی پرسش‌نامه شاخص ناتوانی گردن (Neck Disability Index-Iranian version یا NDI) ثبت گردید. کلیه داده‌ها قبل و يك هفته بعد از مداخله و آستانه درد فشاري با استفاده از آلگومتر قبل، بلافاصله بعد و يك هفته بعد از مداخله جمع‌آوری شد. یافته‌ها: بین ميانگين آستانه درد فشاری در سه زمان (قبل، بلافاصله بعد و يك هفته بعد از مداخله) اختلاف معنی‌داری مشاهده شد (001/0 > P)؛ به طوری که آستانه درد فشاری بلافاصله بعد از مداخله کاهش معنی‌داری را نشان داد (001/0 = P)، اما یک هفته بعد از مداخله نسبت به قبل از آن به طور معنی‌داری افزایش یافت (001/0 = P). ميانگين شاخص ناتوانی و شدت درد افراد، یک هفته بعد از مداخله کاهش معنی‌داري را نشان داد (001/0 > P). نتیجه‎گیری: یک جلسه سوزن زدن خشک در افراد مبتلا به نقاط ماشه‌ای فعال عضله تراپزیوس فوقانی، باعث بهبود معنی‌دار ناتوانی، شدت درد و همچنین، آستانه درد فشاری يك هفته بعد از مداخله می‌گردد.

Title: تغییرات شدت درد افراد مبتلا به سندروم درد پاتلوفمورال (PFPS) به دنبال استفاده از چسب کینزیولوژی و McConnell با استفاده از روش یکسان پیشنهادی McConnell

http://jrrs.mui.ac.ir/index.php/jrrs/article/view/2623

مقدمه: یکی از شایع‌ترین اختلالات اسکلتی- عضلانی ناحیه زانو در افراد بالغ، سندروم دردناک پاتلوفمورال (Patellofemoral pain syndrome یا PFPS) است. روش‌های چسب‌زنی پتلا (Patellar taping) از شیوه‌های درمانی این بیماری محسوب می‌شود. این روش‌ها شامل روش‌های چسب‌زنی McConnell و چسب کینزیولوژی می‌باشد. بنابراین، هدف مطالعه حاضر بررسی تغییرات شدت درد افراد مبتلا به سندروم درد پاتلوفمورال به دنبال استفاده از چسب‌های کینزیولوژی و McConnell با استفاده از روش یکسان پیشنهادی McConnell بود. مواد و روش‌ها: 36 بیمار به صورت تصادفی در سه گروه درمانی چسب McConnell و ورزش، چسب کینزیولوژی و ورزش با روش چسب‌زنی یکسان McConnell جهت اصلاح لغزش خارجی و گروه ورزش به تنهایی (گروه شاهد) به مدت 6 هفته تحت درمان قرار گرفتند. شدت درد قبل و بعد از درمان بیماران اندازه‌گیری شد. یافته‌ها: مقایسه درون گروهی نشان داد که شدت درد بیماران هر سه گروه بعد از پایان درمان به طور معنی‌داری کاهش یافت (001/0 = P برای هر سه گروه). تفاوت معنی‌داری بین گروه McConnell و ورزش با گروه شاهد مشاهده گردید (001/0 = P)، اما این تفاوت بین گروه McConnell با کینزیولوژی (043/0 = P) و گروه کینزیولوژی با شاهد (022/0 = P) معنی‌دار نبود (017/0 < P). نتیجه‎گیری: چسب کینزیولوژی مانند چسب McConnell و با روش چسب‌زنی پیشنهادی McConnell در کاهش درد مؤثر بود.

Title: مقایسه تعادل ایستا و پویای زنان فعال حرکتی دانشگاهی دارای قوس کف پای متفاوت

http://jrrs.mui.ac.ir/index.php/jrrs/article/view/2610

مقدمه: تعادل، یکی از اجزای کلیدی و جدایی‌ناپذیر در فعالیت‌های روزانه و عملکرد‌های ورزشی می‌باشد. شناخت هرچه بهتر و جامع‌تر عوامل تأثیرگذار بر کیفیت تعادل و نحوه اثرگذاری آن‌ها این فرصت را فراهم می‌آورد که به بهبود این عامل پایه حرکتی در تمام طول زندگی کمک کرده و از عواملی که بر آن تأثیر منفی دارد، جلوگیری به عمل آورد. به نظر می‌رسد که وجود ناهنجاری‌های آناتومیکی از قبیل کف پای صاف، گود و ویژگی‌های مورفولوژیکی پا بر آمادگی حرکتی از جمله تعادل افراد تأثیرگذار است. در نتیجه تحقیق حاضر با هدف مقایسه تعادل ایستا و پویای زنان فعال حرکتی دانشگاهی دارای قوس کف پای متفاوت انجام شد. مواد و روش‌ها: تعادل ايستا و پویا 90 ورزشکار زن دانشگاهی در سه گروه قوس کف پایی که شامل کف پای صاف (30 نفر)، کف پای گود (30 نفر) و کف پای طبیعی (30 نفر)، به وسيله دستگاه Stbilometer در حالت ايستاده با چشمان باز (معیار سنجش تعادل ایستا) و قرار دادن یک گام آزمون شونده با پای غالب (معیار سنجش تعادل پویا) ارزیابی شد. همچنین، به منظور تشخیص قوس کف پای صاف، گود و طبیعی از آزمون افت استخوان ناوی با استفاده از روش توصیفی Brady صورت گرفت. تجزيه و تحليل داده‌هاي آماري نیز با استفاده از نرم‌افزار SPSS و از روش آماري تحلیل واریانس يك‌راهه انجام شد. یافته‌ها: در تعادل ایستا بین مساحت کل محدوده جابجایی نوسان بین سه گروه تفاوت معنی‌داری وجود داشت (002/0 = P)؛ در صورتی که در تعادل پویا در مساحت کل محدوده جابجایی نوسان بین سه گروه تفاوت معنی‌داری مشاهده نشد (098/0 = P). نتیجه‎گیری: اگرچه تعادل ایستای افراد مبتلا به ناهنجاری کف پای صاف و گود نسبت به کف پای طبیعی کمتر است، اما در مورد تعادل پویا، تفاوت معنی‌داری بین سه گروه قوس کف پایی مشاهده نشد. بنابراین، می‌توان به افراد مبتلا به کف پای صاف و گود در این زمینه آگاهی داد تا در انجام فعالیت‌های روزانه خود با دقت و توجه بیشتری عمل نمایند.

Title: بررسی هنجار نمره نیزالانس در پسران فارسی زبان 6-4 ساله شهر اصفهان

http://jrrs.mui.ac.ir/index.php/jrrs/article/view/2639

مقدمه: هر ساله گروهی از کودکان مبتلا به VPI (Velopharyngeal insufficiency) جهت بهبود وضعیت تشدید، کاندیدای جراحی‎های ثانویه گفتار می‏شوند، اما نتایج ارزیابی گفتاری که تنها بر اساس قضاوت‎های ادراکی و کیفی درمانگر باشد، پاسخگوی پرسش‌های مربوط به تصمیم‌گیری برای جراحی‏های ثانویه نیست. بنابراین، لازم است قضاوت‎های ادراکی با ارزیابی‎های دستگاهی و عینی تکمیل گردد و اعتبار یابد. ارزیابی‌های دستگاهی به دلیل داشتن پتانسیل برای ارزیابی کمی تشدید خیشومی، مورد توجه می‌باشند. نیزومتر با اندازه‌گیری انرژی آکوستیک دهان و بینی در طی تولید گفتار و محاسبه نمره نیزالانس، یک ارزیابی کمی از خیشومی شدگی ارایه می‌دهد. هدف از انجام مطالعه حاضر، تعیین نمره نیزالانس در پسران 6-4 ساله شهر اصفهان بود. مواد و روش‌ها: این مطالعه از نوع توصیفی- مقطعی بود که در سال 1394 بر روی کودکان 6-4 ساله فارسی زبان شهر اصفهان انجام شد. تمام شرکت کنندگان فارسی زبانی که فرم رضایت‌نامه آگاهانه را تکمیل کرده بودند و بر اساس ارزیابی‌های صورت گرفته، مشکلات صوت، شنوایی، تشدید، گفتار و زبان نداشتند، در مطالعه شرکت نمودند. هنجار نمرات نیزالانس 43 نمونه پسر با استفاده از نسخه فارسی آزمون SNAP (Simplified Nasometric Assessment Procedures) محاسبه گردید. در این مطالعه از دستگاه نیزومتر مدل 6450 استفاده شد. یافته‌ها: میانگین نمره نیزالانس بر اساس آزمون SNAP برای جملات دهانی و خیشومی به ترتیب 16/3 ± 57/11 و 03/7 ± 51/48 بود. نتیجه‎گیری: مطالعه حاضر هنجار نمرات نیزالانس را به عنوان مرجعی در کارهای بالینی از جمله تصمیم‌گیری برای جراحی‌های ثانویه در پسران 6-4 ساله ارایه نمود. با توجه به این که عواملی مانند سن و جنسیت می‌توانند بر نمرات تأثیرگذار باشند، به دست آوردن نمرات نیزالانس در دیگر گروه‌های سنی و جنس مؤنث ضروری به نظر می‌رسد.

Title: تأثیر افزودن برنامه تمرین درمانی بر درمان استاندارد درمانی بر ناتوانی و درد افراد مبتلا به کمردرد حاد غیر اختصاصی: کارآزمايي باليني تصادفی یکسو کور

http://jrrs.mui.ac.ir/index.php/jrrs/article/view/2647

مقدمه: در مورد استفاده از تمرينات فعال در مرحله حاد در افراد مبتلا به کمردرد غیر اختصاصی اختلاف نظر وجود دارد. هدف پژوهش حاضر، مقايسه اثرات درمان استاندارد با و بدون تمرین درمانی در مبتلایان به کمردرد حاد غیر اختصاصی بود. مواد و روش‌ها: تعداد 43 فرد مبتلا به کمردرد حاد غیر اختصاصی به صورت تصادفی به دو گروه آزمون (مانیپولاسیون و تمرین درمانی 21 نفر) و گروه شاهد (مانیپولاسیون 22 نفر) تقسیم شدند. پس از اخذ رضایت‌نامه آگاهانه، اطلاعات جمعیت‌شناسی افراد در فرم مخصوص ثبت گردید. اثرات ضد درد فوري مانيپولاسيون ستون فقرات به وسیله VAS، قبل و بلافاصله بعد از مانيپولاسيون ستون فقرات در هر گروه اندازه‌گیری شد. میزان ناتوانی عملکردی بر اساس نمره پرسش‌نامه Oswestry و درد قبل از اولین جلسه درمان، در پايان آخرين جلسه درمان و يک ماه بعد از اتمام درمان جمع‌آوري شد. یافته‌ها: تعداد 40 نفر مراحل مختلف مطالعه را به طور کامل پشت سرگذاشتند. در هر دو گروه آزمون و شاهد بهبودی معنی‌داری در شدت درد (001/0 > P) و نمره ناتوانی عملکردی (001/0 > P) به دنبال اتمام درمان مشاهده شد، هر چند تفاوت دو گروه در میزان درد (24/0 = P) و ناتوانی عملکردی (42/0 = P) معنی‌دار نشد. از سوی دیگر، میزان کاهش درد بلافاصله بعد از انجام مانیپولاسیون به صورت معنی‌داری کاهش داشت (001/0 > P). نتیجه‎گیری: به نظر می‌رسد که برخلاف بهبود معنی‌دار ناتوانی عملکردی و درد مبتلایان به کمردرد حاد غیر اختصاصی بلافاصله پس از مانیپولاسیون و تمرین درمانی، این بهبودی تفاوت معنی‌داری با بهبودی ناشی از استفاده از مانیپولاسیون به تنهایی ندارد. انجام مطالعات بیشتر با استفاده از برنامه‌های تمرین درمانی مختلف و در انواع مختلف کمردرد توصیه می‌شود.

Title: تأثیر افزایش سن بر سرعت تولید و سرعت گفتار: یک مرور نظام‌مند

http://jrrs.mui.ac.ir/index.php/jrrs/article/view/2617

مقدمه: تغییر در تولید گفتار افراد سالمند دور از انتظار نیست. این تغییر در نتیجه تغییراتی است که در آناتومی و فیزیولوژی سیستم تولید گفتار، بازخوردهای حسی، کنترل حرکتی و پردازش مرکزی گفتار رخ می‌دهد. از آن‌جایی که سالمندی طبیعی، منجر به کندی فرایندهای عصبی می‌شود، انتظار می‌‌رود که بر سرعت گفتار و تولید نیز اثر بگذارد. هدف از انجام این مطالعه مروری نظام‌مند، بررسی تأثیر سن بر سرعت تولید و سرعت گفتار بود. مواد و روش‌ها: کلید واژه‌‌های «Aging و Elderly» در ترکیب با عبارت‌های «Speech rate» و «Articulation rate» در پایگاه‌های اطلاعاتی Google Scholar، PubMed، Scopus و Web of Science در بازه زمانی سال‌های 1959 (زمان آغاز به کار در این حیطه) تا 2016 مورد جستجو قرار گرفت. یافته‌ها: بر اساس معیارهای ورود و خروج، 15 مقاله انتخاب گردید. مقالات یافت شده طبق مقیاس EBM Resources (Evidence-based medicine resources)، در سطح سوم شواهد قرار گرفتند. از بین مقالات بررسی شده، نتایج 4 مقاله با سایر مقالات همخوانی نداشت. اغلب مطالعات نشان دادند که با افزایش سن، سرعت گفتار و سرعت تولید کاهش می‌یابد. نتیجه‎گیری: به دلیل تغییرات آناتومیکی و کاهش ظرفیت‌های شناختی در دوران سالمندی، انتظار می‌رود که سرعت گفتار و سرعت تولید نیز کاهش یابد.
